# Complement-Coagulation Cross-Talk: A Potential Mediator of the Physiological Activation of Complement by Low pH

**DOI:** 10.3389/fimmu.2015.00215

**Published:** 2015-05-06

**Authors:** Hany Ibrahim Kenawy, Ismet Boral, Alan Bevington

**Affiliations:** ^1^Department of Microbiology and Immunology, Faculty of Pharmacy, Mansoura University, Mansoura, Egypt; ^2^Department of Infection, Immunity and Inflammation, College of Medicine, Biological Sciences and Psychology, University of Leicester, Leicester, UK

**Keywords:** complement, coagulation, contact system, alternative pathway, lectin pathway, classical pathway, pH

## Abstract

The complement system is a major constituent of the innate immune system. It not only bridges innate and adaptive arms of the immune system but also links the immune system with the coagulation system. Current understanding of the role of complement has extended far beyond fighting of infections, and now encompasses maintenance of homeostasis, tissue regeneration, and pathophysiology of multiple diseases. It has been known for many years that complement activation is strongly pH sensitive, but only relatively recently has the physiological significance of this been appreciated. Most complement assays are carried out at the physiological pH 7.4. However, pH in some extracellular compartments, for example, renal tubular fluid in parts of the tubule, and extracellular fluid at inflammation loci, is sufficiently acidic to activate complement. The exact molecular mechanism of this activation is still unclear, but possible cross-talk between the contact system (intrinsic pathway) and complement may exist at low pH with subsequent complement activation. The current article reviews the published data on the effect of pH on the contact system and complement activity, the nature of the pH sensor molecules, and the clinical implications of these effects. Of particular interest is chronic kidney disease (CKD) accompanied by metabolic acidosis, in which therapeutic alkalinization of urine has been shown significantly to reduce tubular complement activation products, an effect, which may have important implications for slowing progression of CKD.

## Introduction – The Physiological and Clinical Importance of pH Effects on Complement

It has been known for many years that complement is strongly activated by low pH, especially when pH falls below about 7.1 (1–7). Owing to the tight regulation of arterial blood pH close to the normal physiological value of 7.4 that occurs even under pathological conditions, complement and plasma proteins in major blood vessels are unlikely to be exposed to such low pH. However, at sites of infection or inflammation, a significant localized fall in pH can occur, reaching pH 6 or even lower (8–10). Furthermore, other fluid compartments in mammals, notably the fluid within the lumen of the renal tubule, routinely maintain pH values below 7.1 (Table 1).

**Table 1:** **Summary of intraluminal pH measurements obtained by *in situ* micro-puncture studies in renal tubules of healthy rats**.

Reference	Arterial pH	Early PCT	Late PCT	Near the bend of LoH	Early DCT	Late DCT	CD-proximal end	CD-distal rod	Urine
DuBose et al. (11)	7.33 ± 0.02 [18]	6.98 ± 0.03 [26]	6.72 ± 0.02 [47]	N/A	N/A	N/A	N/A	N/A	5.90 ± 0.43 [9]
DuBose et al. (12)	7.34 ± 0.01 [16]	7.06 ± 0.15 [16]	6.70 ± 0.50 [16]	N/A	6.69 ± 0.15 [16]	6.39 ± 0.04 [16]	N/A	N/A	N/A
DuBose et al. (13)	7.38 ± 0.01 [10]	7.06 ± 0.04 [10]	6.80 ± 0.04 [10]	N/A	6.57 ± 0.07 [10]		N/A	N/A	N/A
Karlmark et al. (14)	7.39 ± 0.01 [12]	N/A	6.68 ± 0.02 [10]	N/A	6.51 ± 0.04 [21]		N/A	N/A	5.67 ± 0.03 [12]
Buerkert et al. (15)	7.37 ± 0.01 [25]	N/A	6.92 ± 0.05 [33]	7.34 ± 0.05 [35]	N/A	6.70 ± 0.07 [16]	6.24 ± 0.0I [14]	5.62 ± 0.01 [14]	5.51 ± 0.21 [10]
Buerkert et al. (16)	7.36 ± 0.01 [12]	N/A	6.87 ± 0.08 [12]	7.39 ± 0.06 [30]	N/A	6.67 ± 0.12 [9]	6.51 ± 0.08 [12]	5.98 ± 0.12 [12]	5.60 ± 0.04 [12]
Winaver et al. (17)	7.34 ± 0.02 [7]	N/A	6.90 ± 0.01 [6]	N/A	N/A	N/A	N/A	N/A	7.84 ± 0.01 [7]

*PCT; proximal convoluted tubule, LoH; loop of Henle, DCT; distal convoluted tubule, CD; collecting duct*.

*Values are mean ± SE; number of observations is shown in parenthesis*.

In healthy individuals, the lumen of the renal tubule is not routinely exposed to plasma proteins (including complement proteins). However, in chronic kidney disease (CKD), leakage of such proteins commonly occurs, resulting in proteinuria. There is now abundant evidence (18) that proteinuria is a major factor driving progression of CKD, and that the leakage of plasma proteins into the tubular lumen triggers an array of pathological changes in proximal tubular epithelial cells (PTEC) (18–20), including hyperplasia and epithelial–mesenchymal transition (EMT), which culminate in end-stage tubulointerstitial fibrosis. The complement system is widely recognized as a key mediator of renal injury (21) and there is mounting evidence that activation of plasma complement proteins leaking into the tubular lumen during proteinuria, followed by strong activation of locally synthesized complement (22) leads to progressive tubulointerstitial damage. Significant amounts of complement activation products are excreted in urine of patients with many forms of proteinuric nephropathy (23) and this excretion of activation products is blunted when metabolic acidosis in these patients is treated with sodium bicarbonate (NaHCO_3_) (23), even though bicarbonate has no long-term effect on proteinuria (23, 24). This implies that, in addition to the well-documented glomerular effects of complement (25), filtered complement, strongly augmented by endogenously expressed tubular complement (22), is activated by the low intratubular pH (Table 1). This may explain the important clinical observation (24) that progression of CKD is significantly slowed in response to therapy with oral alkali (sodium bicarbonate), much of which is excreted into the tubular lumen thus raising intraluminal pH.

While renal complement-activation during metabolic acidosis has traditionally been ascribed to covalent activation of complement C3 by ammoniagenesis (26), more recent direct measurements have failed to substantiate this (7), and direct activation of complement by physiological low pH (4–7) is a more likely explanation, possibly through activation of the alternative pathway (AP) (7, 27) and through pH-sensitive cross-talk between the coagulation (contact) and complement systems.

The current article reviews and compares the basic features of the complement and coagulation systems, cross-talk between these two systems, and the mechanisms whereby low pH may activate complement; in particular, the possibility that low pH is sensed initially by the contact system (intrinsic pathway) and that complement is then activated through contact system-complement cross-talk.

## The Complement and the Coagulation Systems

The complement and the coagulation systems are two closely linked systems that serve a vital role in maintaining homeostasis. Their activities rely on a delicate balance between activator and inhibitor signals of sequential enzymatic reactions that include activation of zymogens and assembly of new proteolytic complexes. Complement is now thought to be involved in several activities besides its role in fighting infections: these include tissue regeneration (28), clearance of debris (29), and pathophysiology of multiple diseases (30, 31). Likewise, the coagulation system plays a role in fighting infections (32) and is implicated in pathophysiology of several diseases besides its role in the maintenance of hemostasis. Furthermore, complex cross-talk between complement and the coagulation system has been described that will be addressed in the current review, particularly with regard to mediating the activation of complement by physiologically attainable low pH.

## The Complement System

Complement, as an integral part of the innate immune system has a major role in defense against invading pathogens. It achieves this through three main strategies; recruitment of immune cells to sites of infection, labeling of the invading pathogens via opsonization for uptake and destruction by phagocytes, and/or direct lysis of susceptible pathogens. Besides bridging innate and adaptive immunity, complement also bridges the immune and coagulation systems. More than 35 proteins, including circulating zymogens, and an array of fluid phase and membrane-bound regulators and cell-bound receptors, participate in complement activities. Three pathways have been recognized for complement activation; the classical pathway (CP), the AP, and the more recently discovered lectin pathway (LP) (33–35). The CP and LP are analogous, differing only in the initiator molecular complexes and the triggering signals (36). Complement C1q in association with two molecules of each of the serine proteases C1r and C1s makes the initiator complex of the CP (37). The C1qrs complex is activated on binding to antigen–antibody complex; however, it can also be activated in an antigen–antibody independent manner by binding to a number of molecules including C-reactive protein (CRP), lipopolysaccharide (LPS), polyanions, viral proteins, pneumolysin (38), and myelin. The LP-initiating complexes are made up of carbohydrate recognition molecules of either mannose binding lectin (MBL), ficolins or collectin-11 (Cl-11) associated with serine proteases, namely, MASP-1, MASP-2, and MASP-3 (39). LP is initiated upon recognition of certain sugar or acetylated sugar patterns decorating surfaces of invading microbes by the broad-spectrum carbohydrate recognition molecules of the LP. Unlike CP and LP, AP activation does not proceed via specific recognition molecules, but occurs instead through an imbalance between activating and inactivating signals acting on a steady state tick over process (40, 41). This kind of imbalance occurs on susceptible surfaces that lack complement regulators or do not support the binding of such regulators that normally occur on host cells. In addition, AP acts as a loop for amplification of signals from the other two pathways (42, 43).

Binding of recognition complexes from either CP or LP to their target structures leads to conformational changes within the molecules that result in activation of the attached serine proteases; C1r/C1s and MASP-1/MASP-2, respectively. The activated serine proteases C1s and MASP-2 cleave C4 into C4a and C4b. C4a is released into the fluid phase, whereas C4b attaches to the target surface. C2 binds to the attached C4b and is cleaved by C1s or MASP-2 releasing C2b into the fluid phase, whereas C2a remains attached to C4b. The resulting complex C4b2a represents the C3-convertase of the CP/LP that then activates C3. The slow and spontaneous hydrolysis of C3 into C3(H_2_O) is considered to provide the flux that maintains the AP activity. The resulting C3(H_2_O) is able to bind to factor B (FB), rendering it susceptible to cleavage by factor D (FD). This produces a limited amount of the fluid phase AP C3-convertase [C3(H_2_O)Bb] that is able to cleave C3 into C3a and C3b. In absence of surface regulators or surfaces that do not support factor H (FH) binding (the main negative regulator of AP), the C3b formed binds to target surfaces and assembles with FB (in presence of FD) the surface AP C3-convertase (C3bBb) (44) that is stabilized by properdin (45, 46).

All of the three complement activation pathways converge in the proteolytic cleavage of C3, where C3 is cleaved into the anaphylatoxin C3a, and the opsonin C3b that binds covalently to the target surface. Binding of several molecules of the C3b to C3-convertases (either from CP/LP or AP) results in the assembly of C5-convertase that cleaves C5 into the powerful anaphylatoxin C5a and the opsonin C5b. Opsonization of target cells with C5b allows, in certain cases, the assembly of terminal complement components C6, 7, 8, and 9 into the membrane attack complex (MAC) that inserts into target membranes forming channels that disrupt membrane function and lead to lysis of target cells (47, 48).

## The Coagulation System

**Figure 1:**
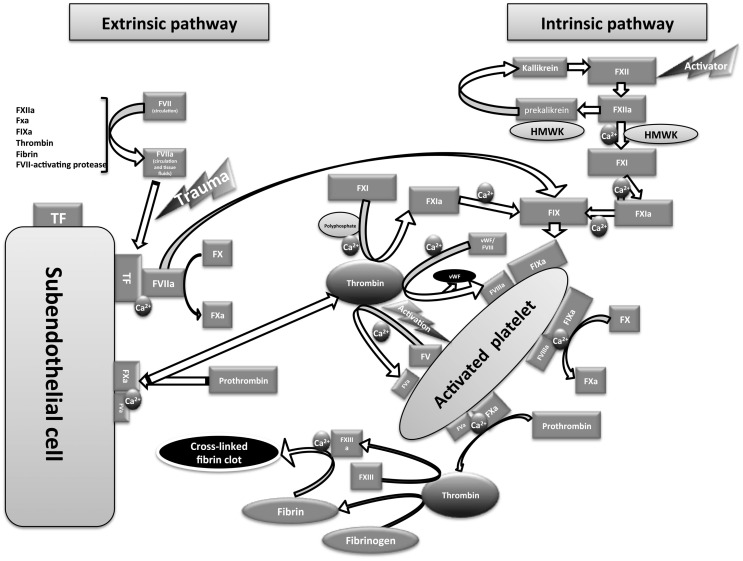
**Diagram showing an overview of coagulation pathways**.

The intrinsic pathway of coagulation was first described by two independent laboratories that proposed the waterfall model (49) and the cascade model (50) of coagulation (Figure 1). Following the discovery of tissue factor (TF) (also called factor III or CD142), the waterfall/cascade models were refined to include the extrinsic pathway. However, these models could not fully explain *in vivo* hemostasis or the normal bleeding tendency in patients lacking some of the early components of the intrinsic pathway [the contact system components: FXII, prekallikrein (PK) and high molecular-weight kininogen (HMWK)]. In addition, it was unclear why patients with functional deficiency of the intrinsic pathway FVIII (hemophilia A) or FIX (hemophilia B) have severe bleeding dysfunction despite the presence of the extrinsic pathway activity. In 1990s, the emergence of the cell-based model of coagulation provided plausible answers to these anomalies (51, 52).

## The Extrinsic Pathway

Blood clotting through the extrinsic pathway of coagulation depends on the interaction between the circulating active form of FVII (FVIIa) and the membrane-bound TF to form a serine protease complex that activates FX into FXa (see Figure 1). FVII is the only coagulation factor found in circulation in active (1%) and inactive forms (53). The mechanism of FVII activation is still unconfirmed; however, autoactivation is suggested to provide the circulating FVIIa (54, 55). Other factors including FXIIa, FXa, FIXa, thrombin, plasmin, and FVII-activating protease also show the ability to activate FVII (56). Under physiological conditions, TF is not accessible to blood components, and only becomes accessible following injury to the endothelial cells lining the blood vessels. TF is expressed in a number of cells, including adventitial cells in the layers surrounding blood vessels. It has been reported that TF is also expressed in a number of activated cells (or cell-derived particles) in the blood, including monocytes, monocyte-derived microparticles, neutrophils, eosinophils, and platelets (57–62). However, intravascular TF exists in an encrypted form that cannot interact with FVIIa unless decrypted, possibly through the enzymatic activity of protein disulfide isomerase (63–65). Intravascular TF is thought to be involved in thrombosis (a pathological form of coagulation) rather than normal hemostasis (57, 59, 66). The extrinsic pathway is believed to be the only physiological trigger of coagulation *in vivo* that is activated immediately upon blood vessel injury (67).

## The Intrinsic Pathway and the Contact System

The intrinsic pathway of coagulation is triggered upon the activation of the first component of the contact system “FXII” into FXIIa by an activator surface that is usually a negatively charged surface (68). The *in vivo* physiological activators of FXII are still unclear; however, platelet-derived polyphosphate (54, 69, 70) and mast cell heparin (71) are suggested to be contributory physiological activators of the contact system. Other known activators of FXII include extracellular RNA (72), DNA (73), collagen, Kaolin (74), dextran sulfate (75, 76), oversulfated chondroitin sulfate (77), glass, and plastic. FXIIa activates the second component of the contact system – PK into kallikrein. PK circulates with the cofactor HMWK (the third contact system component). The resulting kallikrein promotes the activation of additional FXII molecules via a positive feedback loop (Figure 1). These first two steps in contact system activation do not require the presence of calcium ions. The resulting FXIIa in the presence of calcium ions, phospholipids (phosphatidylserine provided by activated platelet surfaces), and the cofactor HMWK activates FXI into FXIa (78). FXIa then activates FIX into FIXa in the presence of calcium ions and phospholipids. FIXa forms (with the cofactor FVIIIa, in presence of calcium ions and phospholipids) a serine protease “Tenase” that activates FX into FXa. The resulting FXa either from the extrinsic or the intrinsic pathway forms, with the cofactor “FVa,” a serine protease called “prothrombinase” that activates prothrombin (FII) into thrombin (the common pathway). The resulting thrombin then acts on fibrinogen, releasing fibrin monomer that is cross-linked in the presence of FXIIIa to form a stable fibrin polymer clot. Besides the procoagulant activity, kallikrein can cleave HMWK to release the proinflammatory peptide “bradykinin” (kallikrein-kinin pathway) that acts on multiple target cells with the production of inflammatory mediators that include prostacyclin, prostaglandins, leukotrienes, endothelial-derived hyperpolarizing factor, and nitric oxide (79). By generation of bradykinin, the contact system plays an additional role, promoting other immune defense mechanisms during infection [for review, see Ref. (80)].

Growing evidence suggests that the contact system has surprisingly little impact on physiological hemostasis. Deficiency of FXII in humans and other animal species is not associated with deficient hemostasis. Moreover, non-mammalian vertebrates and cetaceans do not have FXII (81). On the other hand, it is strongly suspected that the contact system is associated with thrombosis (82). Deficiency of the contact system is protective against development of thrombosis and stroke (32, 83, 84). It is now believed that the mechanisms of physiological hemostasis are different from those of thrombosis (82) and thrombosis can be triggered via the activation of FXII by platelets and erythrocyte-derived microparticles (85). Current therapeutic strategies in thrombosis focus on targeting the contact system, hence affording protection from thrombosis or embolism without interfering with the hemostatic capacity. In view of the apparent links between the contact system and the activation of complement at low pH (discussed below), the contact system might also be a suitable therapeutic target in blocking the detrimental effects of complement activation under acidic conditions.

## The Cell-Based Model of Coagulation

A more complete understanding of *in vivo* coagulation requires the cell-based model of coagulation, in which cell surfaces play a role distinct from providing the phospholipids required for assembly of protease complexes via platelet surfaces. The initiation step is believed to occur continuously in the extravascular tissue fluids and lymph, into which coagulation factors such as FVII, FIX, FX, prothrombin, and other low molecular-weight coagulation factors can diffuse from blood (51). In this step, FVIIa binds to TF on TF-bearing cells to form TF/FVIIa complex that activates FX to FXa. The resulting FXa activates prothrombin to thrombin before being rapidly inactivated by tissue factor pathway inhibitor (TFPI) and antithrombin III (86, 87). At the same time, TF/FVIIa complex activates FIX into IXa (88) that is required in further steps. Thus, this initiation step provides a continuous supply of trace amounts of extravascular thrombin that is required for further steps in the event of blood vessel injury. If such injury occurs, the following amplification and propagation steps will be triggered, involving blood coagulation factors and platelets that cannot normally diffuse from blood vessels into surrounding tissues. This occurs when these come into contact with the thrombin generated from the initiation phase (as described above), and the TF-bearing cells and subendothelial collagen.

Subsequently, platelets will be activated at the injury site by thrombin and collagen (89), releasing FV that becomes activated by thrombin on the platelet surface (see Figure 1). At the same time, thrombin will release FVIII from von Willebrand factor (vWF)-FVIII complex and activate it to FVIIIa. During this amplification step, the activated platelets are covered with the cofactors FVIIIa and FVa. The amount of thrombin generated at this stage is not sufficient to drive clot formation; however, it is very important for the amplification of the procoagulant signal.

In the subsequent propagation step, FIXa produced either from TF/FVIIa complex or from FXIa generated via thrombin action on FXI [in the presence of polyphosphate (90)] will bind to FVIIIa on platelets in the presence of calcium ions to form tenase that is 50 times more efficient than TF/FVIIa complex in FX activation (87), thus releasing large amounts of FXa. This FXa assembles with FVa and calcium to form prothrombinase on the activated platelet surface that will generate copious amounts of thrombin, which drive fibrin clot formation. Accordingly, TF/FVIIa appears to be the primary physiological trigger of *in vivo* coagulation (67) and contact system components (FXII, PK, and HMWK) do not seem to have a major role.

## Cross-Talk between Complement and Coagulation Systems

The complement and coagulation systems share a number of common features. Activation of both systems leads to conversion of zymogens and assembly of proteolytic complexes that are mostly serine proteases of high-substrate specificity. Interactions between complement and coagulation systems have been described in a number of publications. For example, some complement regulators such as complement C1 inhibitor are involved in regulation of the contact system (91), and serine proteases from either of the two systems may act on substrates from the other system. For this reason, severe trauma and acute blood loss are not only associated with disseminated intravascular coagulopathy (DIC) but also with massive complement activation. This generates the potent anaphylatoxins C3a and C5a, and these may in their turn intensify coagulation (92–94). Activated platelets, which are critical participants in coagulation, can also activate both the CP (95) and the AP (96, 97); however, the physiological impact of this activation is still unknown, although complement activation products are known to activate platelets (97), which may lead to a positive feedback loop.

Thrombin generated from the coagulation system can activate complement C3 and C5 independent of the established complement activation pathways (98). Furthermore, C3 and C5 activation can proceed independent of each other (98). Similarly, Amara et al. (92, 99) reported the cleavage of C3 and C5, with the generation of C3a and C5a, respectively, by the coagulation factors FIXa, FXa, FXIa, and plasmin (100) independent of the known complement activation pathways. Thrombin and plasmin have been suggested to activate complement during liver regeneration in the absence of C4 and AP activity (28). In addition, FXIIa has been shown to activate the CP of complement via activation of C1qrs complex (101). Surprisingly, fluid phase activation of FXII by oversulfated chondroitin sulfate activated not only the contact (Kallikrein-Kinin) system but also C3 and C5 in the presence of EDTA (77). EDTA is a well-known inhibitor of complement via all of the known activation pathways by sequestering the divalent cations (Ca^2+^ and Mg^2+^) necessary for complement activation. Thus, C3 and C5 activation can proceed by a mechanism that is independent of the known C3- and C5-convertases. Depletion of FXII from plasma abolished this activation without affecting the normal complement activity; and reconstitution of depleted plasma with purified FXII restored complement activation (77).

However, not all such effects involve FXII: the use of aprotonin – a protease inhibitor of kallikrein and plasmin (but not of FXII) – inhibited C5 activation by oversulfated chondroitin sulfate (77). Furthermore, plasminogen-depleted plasma also failed to induce C5a production by oversulfated chondroitin sulfate (77). Thus, it seems that the contact system activity – not exclusively the individual activities of FXIIa or kallikrein – may drive complement C3 and C5 activation through generation of plasmin that has previously been reported to cleave C3 and C5 (77, 92, 100).

Wiggins et al. (102) showed that purified rabbit kallikrein was able to generate from rabbit C5 an activity that was chemotactic for rabbit neutrophils, suggesting that Kallikrein may cleave C5. Besides that, kallikrein was shown to play a role similar to factor D in cleaving C3bB, generating the AP C3-convertase C3bBb. However, this activity required the presence of divalent cations (103).

Conversely, complement components have been shown to influence coagulation activity in multiple ways. The key enzyme of LP activation (MASP-2) is able to generate thrombin through direct cleavage of prothrombin (104). Likewise, the terminal complement component complex C5b–9 has similar activity toward prothrombin even in absence of FV (105). Moreover, both the sublytic MAC and the cytolytically inactive terminal complement complex exhibit procoagulant activity mediated via the induction of TF expression by endothelial cells (106). The anaphylatoxin C5a promotes procoagulant activity by several actions on cells. C5a induces the upregulation of TF expression by endothelial cells (107) and by neutrophils (108). In addition, C5a induces the switch of mast cell and basophil activities from profibrinolytic to prothrombotic through the upregulation of plasminogen activator inhibitor-1 (PAI-1) (109). Interaction between the two membrane receptors TF and complement C5a receptor (C5a R) further suggests a cross-talk between the two systems (108). Accordingly, there is considerable evidence for two-way communication between complement and coagulation system components, influencing the activities of both systems.

## Complement and Contact System Activities at Acidic pH

Several *in vitro* studies have demonstrated potent activation of complement activity, at acidic pH values, some of which are in the physiologically relevant range shown in Table 1 (1–7). However, care is needed in interpreting these effects for two reasons: first because *in vivo* the low pH may have multiple sites of action (as in the case of renal tubules or inflammatory foci); and second because not all such effects observed *in vitro* may be directly physiologically relevant because they require extremely low pH or non-physiological temperature. For example, Hammer et al. (1) reported that acidification of serum or C5 and C6 to pH 6.4 at 0°C followed by neutralization was associated with complement activation that led to lysis of non-sensitized red blood cells in presence of terminal complement components C7, C8, and C9. They attributed the observed activity to the formation of a complex between C5 and C6 similar in activity to that of C5b6 generated via the AP or the CP. This complex was thought to be formed as a result of C6-mediated cleavage of C5 α-chain aided by low pH, which changed the tertiary structure of either or both of C5 and C6. However, the physiological significance of this complex is uncertain, as it was unstable at physiological temperature (37°C).

Low pH may also exert multiple effects through the AP. AP hemolytic activity on erythrocytes from paroxysmal nocturnal haemglobinuria (PNH) patients (110, 111) and rabbit erythrocytes was enhanced at pH 6.4 compared to pH 7.4 (2), which forms the basis of Ham’s test used in diagnosis of PNH (110). They explained the enhancement of activity through the increased formation of the two C3-convertases; C3(H_2_O)Bb and C3bBb, in addition to the enhanced binding of FB and C5 to C3b deposited on erythrocytes at pH 6.4. At the same time, the inhibitory effect of CR1 and factor I (FI) was also diminished at this pH. Similarly, Peake et al. (7) suggested that maximal complement deposition on cultured PTEC occurred via the AP at acidic pH.

Complement activation under mildly acidic conditions has also been attributed to human CRP via the CP (3). CRP is known to trigger CP activation upon interaction with phosphocholine-containing or polycationic agents. However, even in absence of these agents, CRP has been reported to activate complement, with optimal activity at pH 6.3. Furthermore, Hammond et al. (112) showed that CRP was able to interact with FH, which may be a way of partially regulating the enhanced complement activity mediated by CRP at low pH. However, it should be noted that this interaction of CRP with FH required more acidic pH (5.2–4.6).

## Contact System-Complement Cross-Talk as a Mediator of the Effect of Low pH on Complement

Apart from the evidence cited above, relatively little is known of direct effects of mildly acidic pH on complement proteins or complement regulatory proteins. An alternative explanation of the activation of complement by low pH is that pH is initially sensed by component(s) of the contact system and this then leads indirectly to complement activation through complement-coagulation cross-talk. In the study involving CRP cited above (3), complement activation occurred in glass tubes, but not in polypropylene tubes. Addition of Kaolin to polypropylene tubes restored complement activation, suggesting a requirement for the presence of negatively charged surfaces to support the pH-dependent CRP-mediated complement activation (3). These authors attributed these effects of low pH to the conformational changes that CRP underwent at this pH. However, a possible alternative explanation is that the contact system is involved in this CRP-mediated complement activation at mild acidic pH (3), as activation occurred only in the presence of negatively charged surfaces, conditions that activate the contact system as well.

Further evidence in support of a role for the contact system in mediating the activation of complement by low pH (through complement-coagulation cross-talk) comes from the observation that low pH results in accumulation of FXIIa as well as complement activation products. Generation of complement activation products C3a and C5a was observed by Emeis et al. (4) upon acidification of blood with either HCl or lactic acid. This complement activation was attributed to the effect of acidic pH itself, not to the lactate anion, as addition of lactate at control (non-acidic) pH had no such effect. Likewise, respiratory acidosis of blood was also associated with increase in C3a and C5a levels. Lactate acidosis could activate not only C3 and C5 but also more distal steps in the complement system, with the formation of the soluble terminal complement complex (sC5b–9) either in adults or neonates (113). In addition, Sonntag et al. (5, 6) showed that acidification of blood and plasma with lactic acid was associated with dose-dependent increase in complement C5a and contact system FXIIa generation, even in the absence of cellular components. Similarly, Renaux et al. (114) and Thomas et al. (115) reported increased contact system activity with increased kallikrein activity and bradykinin formation during hemodialysis upon lowering the pH of diluted plasma from pH 7.4 to 7.1.

In addition to the *in vitro* studies, parallel activation of complement and the contact system has also been observed *in vivo* in conditions associated with tissue acidosis, for example, in cases of myocardial infarction, shock, and perinatal asphyxia, where the anaphylatoxins C3a and C5a, and FXIIa levels were elevated (5, 6, 116, 117).

The molecular basis of this apparent strong relation between complement activation and contact system activation under mildly acidic pH conditions, where the contact system is probably the driving force for the complement activation, is still unclear. The pH sensor molecule(s) involved are unknown. A highly pH-sensitive plasma protein is the histidine proline-rich glycoprotein [HPRG – reviewed in Ref. (118)] in which much of the pH sensitivity is attributable to the “histidine rich” region of the molecule. Even though HPRG is known to associate with complement proteins (118, 119), no direct acid-dependent activation of complement by this molecule has ever been demonstrated. However, an interesting potential link with the contact system arises from the observation that HPRG and HMWK show about 50% sequence identity in their “histidine rich” region (118), indicating a possible direct pH-sensing role by HMWK.

In view of the apparent links between acid-dependent complement activation and the progression of CKD that were reviewed at the start of this article, the molecular basis of this effect of low pH, and the involvement of possible pH sensing in the contact system, merits further investigation; particularly as conventional therapy for correction of low pH by administering oral sodium bicarbonate to CKD patients may carry with it cardiovascular risks associated with sodium loading. Selective inhibition of the contact system (which may be possible without impairing hemostasis) might be a suitable alternative therapeutic target. However, before this possibility is pursued, two important points about the activation of complement at low pH remain to be clarified:

First, if the complement activation ultimately arises from complement-coagulation system cross-talk, with the cleavage of C3 and C5, by FIXa, FXa, FXIa, and plasmin (100), it needs to be confirmed that these proteases are still active at the relevant low pH. If this is important in the renal tubular lumen during proteinuria, it also needs to be shown that the failure of glomerular permselectivity during proteinuria is sufficient to allow the relevant contact system components (and not just complement) to leak into the acidic renal tubular lumen.

Second, it needs to be confirmed that the effect of elevated pH *in vitro*, or the therapeutic effect of alkalinizing the tubular lumen with bicarbonate therapy *in vivo* (24) arises from inhibiting the contact system, rather than through blocking the other proposed effects of low pH on complement, such as acid-induced amplification of spontaneous AP activity or enhanced binding of FB and C5 to C3b (2).

## Conflict of Interest Statement

The authors declare that the research was conducted in the absence of any commercial or financial relationships that could be construed as a potential conflict of interest.
